# Caveolin-1 gene therapy inhibits inflammasome activation to protect from bleomycin-induced pulmonary fibrosis

**DOI:** 10.1038/s41598-019-55819-y

**Published:** 2019-12-23

**Authors:** Xin Lin, Michael Barravecchia, R. Matthew Kottmann, Patricia Sime, David A Dean

**Affiliations:** 1 Department of Pediatrics, School of Medicine and Dentistry, University of Rochester, Rochester, NY 14642 USA; 2 Department of Medicine, School of Medicine and Dentistry, University of Rochester, Rochester, NY 14642 USA

**Keywords:** Respiration, Molecular medicine

## Abstract

Idiopathic pulmonary fibrosis (IPF) is a devastating and fatal disease and characterized by increased deposition of extracellular matrix proteins and scar formation in the lung, resulting from alveolar epithelial damage and accumulation of inflammatory cells. Evidence suggests that Caveolin-1 (Cav-1), a major component of caveolae which regulates cell signaling and endocytosis, is a potential target to treat fibrotic diseases, although the mechanisms and responsible cell types are unclear. We show that Cav-1 expression was downregulated both in alveolar epithelial type I cells in bleomycin-injured mouse lungs and in lung sections from IPF patients. Increased expression of IL-1β and caspase-1 has been observed in IPF patients, indicating inflammasome activation associated with IPF. Gene transfer of a plasmid expressing Cav-1 using transthoracic electroporation reduced infiltration of neutrophils and monocytes/macrophages and protected from subsequent bleomycin-induced pulmonary fibrosis. Overexpression of Cav-1 suppressed bleomycin- or silica-induced activation of caspase-1 and maturation of pro-IL-1β to secrete cleaved IL-1β both in mouse lungs and in primary type I cells. These results demonstrate that gene transfer of Cav-1 downregulates inflammasome activity and protects from subsequent bleomycin-mediated pulmonary fibrosis. This indicates a pivotal regulation of Cav-1 in inflammasome activity and suggests a novel therapeutic strategy for patients with IPF.

## Introduction

Idiopathic pulmonary fibrosis (IPF) is a disease characterized by the progressive and fatal destruction of lung architecture caused by acute lung injury with subsequent scar formation that ultimately leads to respiratory failure^1,2^. Usually, IPF occurs in adults between 50 and 70 years of age, and the median survival rate is ∼3 yr after diagnosis^3,4^. Hallmarks of IPF include increased deposition of collagen and other extracellular matrix proteins and the accumulation of scar tissue in the lung interstitium, resulting from damage to the alveolar epithelium and accumulation of inflammatory cells^1,5^. Although a great deal of investigation has been aimed at the treatment of IPF, there are only limited treatment options^6,7^.

The importance of inflammation and the innate immune response in fibrogenesis and the pathogenesis of IPF has been well established, although some doubts persist as to whether inflammation is necessary to cause fibrosis due to the failure of anti-inflammatory medications to treat IPF patients^1,5,8^. The Nacht Domain-, Leucine-Rich Repeat-, and PYD-containing Protein 3 (NLRP3) inflammasome has recently been found to play a critical role in mediating cell response to bleomycin- and silica-induced pulmonary inflammation and fibrosis^2,9,10^. The inflammasome regulates and activates the innate immune response through the secretion of pro-inflammatory cytokines interleukin −1 beta (IL-1β) and interleukin −18 (IL-18) and is involved in a variety of lung diseases, such as asthma and chronic obstructive pulmonary disease (COPD)^11^. Key components of the inflammasome are a sensor protein, the adapter protein ASC, and the inflammatory protease caspase-1^12^. Once activated, the assembled inflammasome mediates caspase-1 cleavage, which in turn induces pro-IL-1β and pro-IL-18 to be cleaved to their active forms and secreted from cells. Deficiency of sensors NLRP3 or ASC, or inhibition of caspase-1, decreases collagen synthesis, reduces α-smooth muscle actin expression, and fails to generate fibrosis after exposure to bleomycin^13,14^.

Caveolae are invaginations in the plasma membrane of cells that regulate many cellular functions, including membrane trafficking, endocytosis, lipid metabolism, signal transduction, cellular proliferation, and apoptosis^15^. Caveolin-1 (Cav-1) is a major protein component of caveolae and is highly expressed in a variety of cell types in the lung, including alveolar epithelial type I cells, endothelial cells, fibroblasts, and leukocytes^16^. It is associated with the regulation of numerous signaling pathways, including mitogen-activated protein kinase (MAPK) and phosphoinositide 3-kinase (PI3K) pathways, resulting in cell growth suppression and induction of apoptosis. Recent findings indicate that Cav-1 contributes to the regulation of fibrosis through regulation of TGF-β signaling^17^, ECM production^18^, and inhibition of inflammation^19–21^. Cav-1–deficient mice develop spontaneous fibrosis with markedly smaller individual alveolar volumes, thickened alveolar walls and increased collagen and extracellular fibrillar deposition, as well as additional immune defects^22–25^. Further, greatly reduced levels of Cav-1 have been found in the lungs of IPF patients^26^. It has been previously reported that bleomycin-induced lung fibrosis can be attenuated in mice following overexpression of active Cav-1 by adenovirus or Cav-1 scaffolding domain peptide^26,27^. Although the majority of studies have demonstrated that Cav-1 has an anti-fibrotic effect in the lung^22–24,26,28^, a recent study has reported that Cav-1^-/-^ mice showed reduced fibrosis after bleomycin instillation^29^. Thus, the exact nature and mechanism of Cav-1’s role in pulmonary fibrosis remain open.

Research from our laboratory has demonstrated that electroporation can be used to efficiently deliver DNA to the lungs of living animals without injury or inflammation^30–32^. In this study, we show that electroporation-mediated gene transfer of plasmids expressing Cav-1 can protect from bleomycin-induced pulmonary fibrosis through downregulation of inflammasome activity in the lung epithelium. We investigated inflammasome activity in lung tissues from IPF patients and control subjects and report for the first time that Cav-1 regulates inflammasome activity in lung epithelial cells.

## Results

### Cav-1 is downregulated in alveolar epithelial type I cells in a bleomycin-induced pulmonary fibrosis mouse model

**Figure 1:**
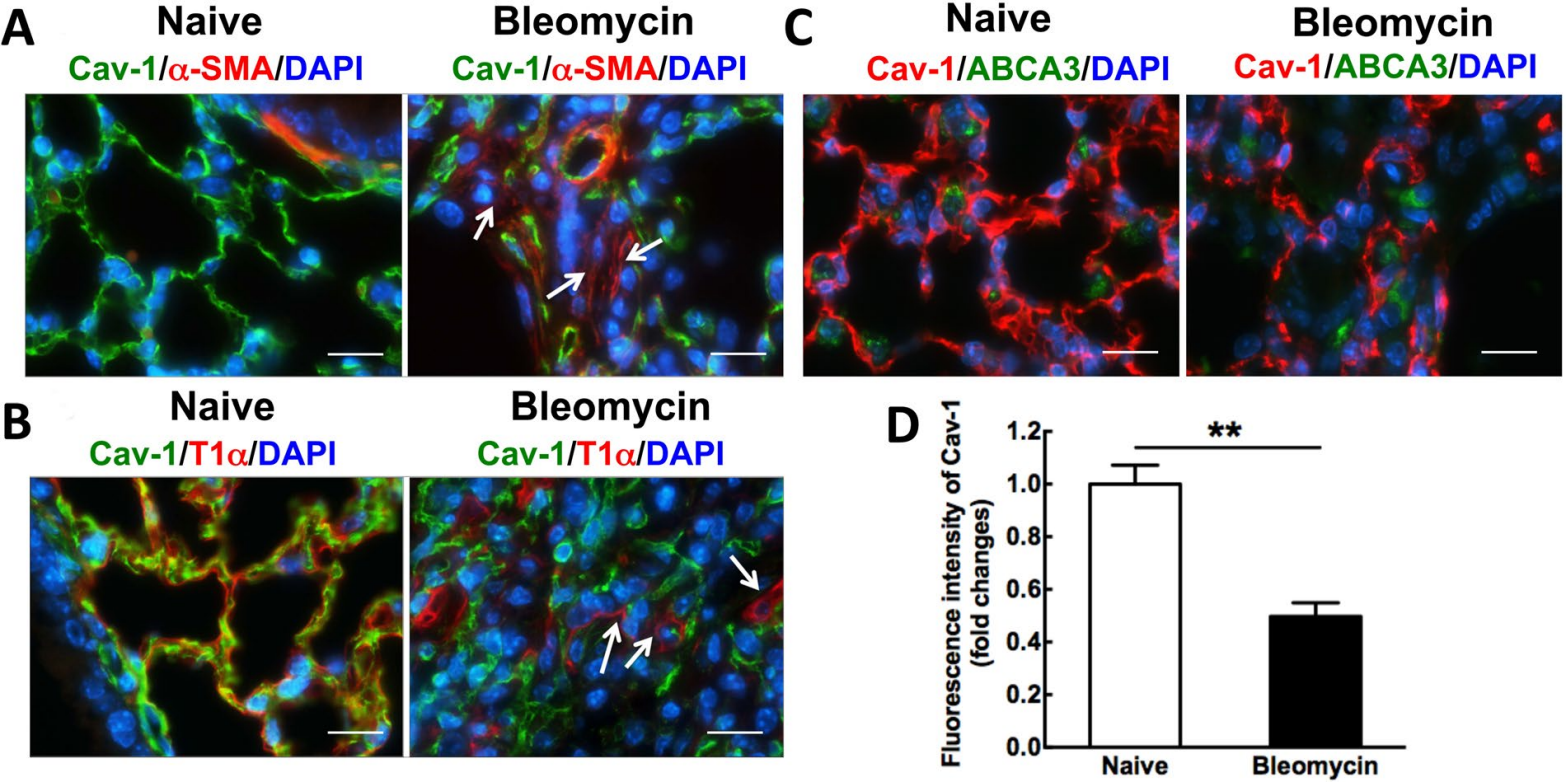
Cav-1 expression is decreased in bleomycin-induced pulmonary fibrosis, primarily, in alveolar epithelial type I cells after bleomycin administration for 21 days. (**A**) Immunofluorescence analysis of Cav-1 expression in lung tissue sections (Cav-1, green; nucleus, blue; α-SMA, red). (**B**) Immunofluorescence analysis of T1α, an epithelial type I cell marker, and Cav-1 expression in bleomycin-induced fibrosis tissue sections, compared to normal lungs (Cav-1, green; nucleus, blue; T1α, red). (**C**) Immunofluorescence analysis of ABCA3, an epithelial type II cell marker, and Cav-1 expression in bleomycin-induced fibrosis tissue sections, compared to normal lungs (Cav-1, red; nucleus, blue; ABCA3, green). Arrows indicate that Cav-1 expression is decreased in α-SMA-positive cells or epithelial type I cells. Scale bar: 10 μm. (**D**) Fluorescence intensity of Cav-1 was quantified using Image J software in the tissue sections (n = 8). Statistical analysis was by unpaired t test (mean ± SD; n = 4 mice per group), **P < 0.01.

Although others have reported that Cav-1 expression is reduced in samples of both experimental animal models of pulmonary fibrosis and patients with IPF^26,28,33^, we determined in which cell type Cav-1 was altered in our bleomycin-mediated pulmonary fibrosis mouse model (Fig. 1). The reduction of Cav-1 expression was measured by immunofluorescence staining. Cav-1 expression is significantly decreased in the fibrotic area, which appears as increased expression of alpha smooth muscle actin (α-SMA), a hallmark of lung fibrosis, in alveoli after bleomycin administration for 21 days, compared to control mice (Fig. 1A). To determine cell type-specific loss of Cav-1 expression in bleomycin-induced mouse lungs, we performed co-staining of Cav-1 with T1α, an epithelial type I-specific marker, or ABCA3, an epithelial type II-specific marker. The cells positive for T1α show markedly reduced Cav-1 expression compared with control mice (Fig. 1B), whereas the cells positive for ABCA3 do not co-express with Cav-1 although a 60% reduction of Cav-1 expression is observed in bleomycin-injured lungs 21 days after administration, compared to naïve mice (Fig. 1C,D). These results indicate that expression of Cav-1 is decreased primarily in epithelial type I cells in bleomycin-induced fibrotic mouse lungs.

### Gene transfer of Cav-1 protects from bleomycin-mediated pulmonary fibrosis

**Figure 2:**
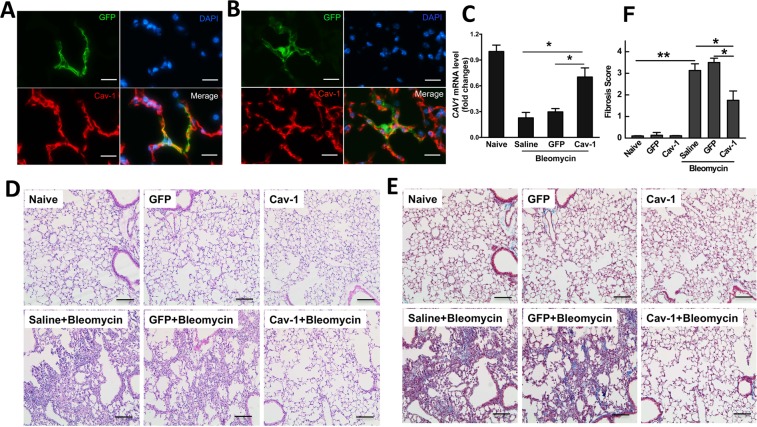
Electroporation-mediated gene transfer of Cav-1 protects from bleomycin-induced pulmonary fibrosis. 100 μg of plasmid expressing GFP-tagged Cav-1 driven by the UbC promoter in 50 μl was delivered intratracheally to C57BL/6 mice and electroporated at 200 V/cm using 8 pulses of 10 msec duration. One day later, bleomycin (2 Unit/kg) was given to the lungs and the lungs were harvested at 21 days and subjected to (**A**) immunofluorescence staining to determine co-localization of GFP with Cav-1 in healthy mouse lungs or (**B**) bleomycin-injured lungs (GFP, green; nucleus, blue; Cav-1, red) (Scale bar: 10 μm), (**C**) Real-time PCR of left lobe, (**D**) H&E staining and (**E**) Masson trichrome staining of right lung tissue. One representative example out of four is shown. Scale bar: 100 μm. (**F**) Fibrosis score was evaluated by the Ashcroft scale in the tissue sections (n = 4). Statistical analysis was by one-way ANOVA (mean ± SEM; n = 4 mice per group), *P < 0.05 or **P < 0.01.

Given previous studies have shown that bleomycin-induced lung fibrosis can be attenuated in mice following over-expression of active Cav-1 by adenovirus or Cav-1 scaffolding domain peptide^26,27^, we determined whether electroporation-mediated gene transfer of Cav-1 provided efficient protection against bleomycin-induced pulmonary fibrosis without side effect. Mice were electroporated with a plasmid expressing Cav-1 or GFP vector control 1 day prior to bleomycin administration. Lung tissues were harvested 3 weeks after injury and fibrotic formation was analyzed (Fig. 2). To ensure that our gene transfer approach and delivered transgenes could be expressed for sufficient time in this model, plasmids expressing GFP-tagged Cav-1 driven by the long-acting UbC promoter were electroporated into mouse lungs, and 3 weeks later, GFP and Cav-1 expression were analyzed in mouse lungs (Fig. 2A). GFP-Cav1 expression, as determined by co-localization of GFP- and Cav-1 staining was detected 3 weeks after electroporation in both healthy mice and those injured with bleomycin, demonstrating good duration of expression in healthy and injured lungs (Fig. 2B). As shown in Fig. 2C, bleomycin administration decreased mRNA levels of Cav-1 in mice that received control GFP plasmid. By contrast, delivery of a plasmid expressing Cav-1 inhibited bleomycin-induced loss of Cav-1 expression. Alveolar architecture was retained and no differences were seen among the naïve, GFP-vector control, and Cav-1 groups without bleomycin challenge (Fig. 2D,E). By contrast, both saline and vector control groups in bleomycin-treated mice showed lung fibrosis with marked disruption of the alveolar space, increased wall thickening, and inflammation. However, in bleomycin-treated mice that had received the Cav-1 plasmid, lungs showed much less destruction of the alveolar space and fewer infiltrated inflammatory cells. An Ashcroft fibrosis score showed a 60% decrease in the Cav-1 group compared with saline or the GFP control groups (Fig. 2F).

**Figure 3:**
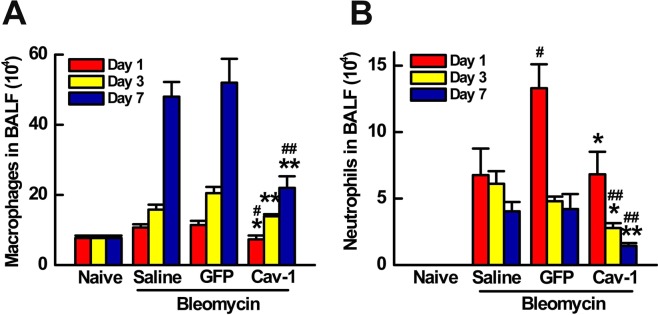
Gene transfer of Cav-1 reduces neutrophils and macrophages in BALF of bleomycin-challenged mice. (**A**) Alveolar macrophages and (**B**) neutrophils were quantified in BALF at days 1, 3, and 7 after bleomycin instillation. Cav-1 gene delivery prior to bleomycin decreased macrophage and neutrophil infiltration at all times compared to mice receiving control GFP plasmid and bleomycin. Statistical analysis was by one-way ANOVA (mean ± SEM; n = 5 mice per group). *P < 0.05, *P < 0.01 *vs* vector control; ^#^*P* < 0.05, ^# #^ *P* < 0.01 *vs* bleomycin only.

To determine the effects of Cav-1 gene transfer on acute lung inflammation, the inflammatory cell response was evaluated in bronchoalveolar lavage fluid (BALF) after intratracheal bleomycin administration at day 1, 3 and 7. Alveolar macrophages in BALF were increased five-fold over 7 days in mice after bleomycin challenge compared with naïve mice. As can be seen, transfer of GFP before bleomycin instillation resulted in no change in macrophage infiltration compared to bleomycin only mice. By contrast, transfer of the Cav-1 plasmid significantly decreased the number of macrophages in BALF to 7.3 ± 1.1, 14 ± 0.6, or 22 ± 3.3 (× 10^4^/ml), compared to 11.4 ± 1.2, 20 ± 1.8, or 52 ± 6.8 (× 10^4^/ml) of the control GFP at days 1, 3, and 7, respectively (Fig. 3A). As shown in Fig. 3B, neutrophil recruitment in BALF was observed within one day of bleomycin administration, and persisted over 7 days, compared to naïve mice. Gene transfer of Cav-1 significantly reduced bleomycin-induced neutrophil infiltration compared with bleomycin only and control groups. Collectively, these results demonstrate that electroporation-mediated gene transfer of Cav-1 can protect against subsequent bleomycin-induced pulmonary fibrosis and abrogate pulmonary inflammatory response to bleomycin injury.

### Gene transfer of Cav-1 suppresses bleomycin-induced inflammasome activation in mouse lungs

**Figure 4:**
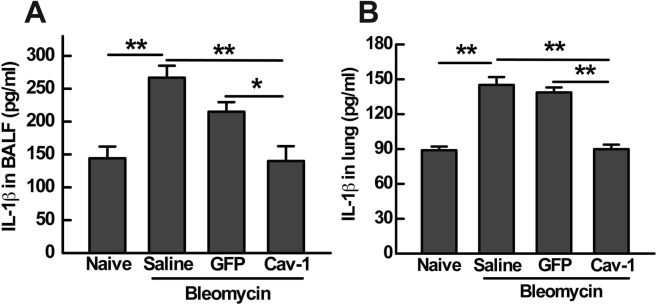
Gene transfer of Cav-1 decreases IL-1β production in both BALF and lungs of bleomycin-challenged mice. IL-1β production in BALF (**A**) and lung (**B**) was analyzed at day 1 after bleomycin administration measured by ELISA. Statistical analysis was by one-way ANOVA (mean ± SEM; n = 5), *P < 0.05 or **P < 0.01.

Increasing evidence suggests that activation of the inflammasome leads to pulmonary inflammation and fibrosis^2,9^. Since bleomycin-induced acute lung injury may activate the inflammasome to facilitate the secretion of pro-inflammatory cytokines, including the release of active IL-1β^2^, we hypothesized that activation of the inflammasome could be associated with the protective effects of Cav-1gene transfer on bleomycin-induced fibrosis. One day after bleomycin administration, IL-1β production was detected in both BALF and in lung homogenates by ELISA. As shown in Fig. 4, IL-1β production in response to bleomycin was enhanced two-fold compared with naïve mice. Transfer of the control GFP plasmid one day after bleomycin instillation resulted in no change in secretion of IL-1β. As we expected, gene transfer of Cav-1 significantly reduced bleomycin-induced IL-1β production to 140 ± 22.5 pg/ml in the BALF (Fig. 4A) or 89.9 ± 3.9 pg/ml in the lung (Fig. 4B), compared to 215 ± 14.5 or 138.7 ± 4.3 pg/ml of the empty GFP plasmid, respectively.

**Figure 5:**
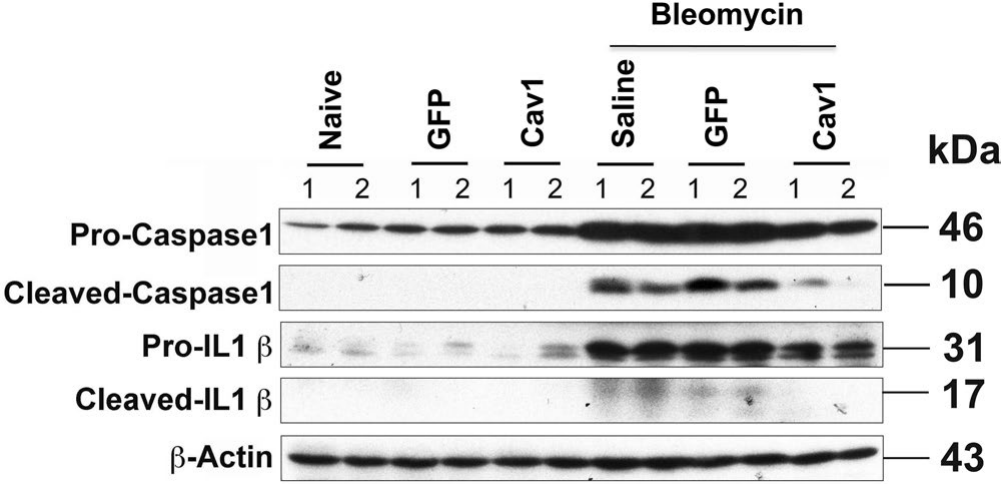
Gene transfer of Cav-1 suppresses bleomycin-induced inflammasome activation in mouse lungs 21 days after bleomycin administration. 100 μg of plasmid in 50 μl was delivered intratracheally to C57BL/6 mice and electroporated at 200 V/cm using 8 pulses of 10 msec duration. One day later, bleomycin (2 Unit/kg) was given to the lungs and the lungs were harvested at 21 days and subjected to western blot. Cropped blots were used in this figure. Original full-length blots are presented in Supplementary Figure S1.

To further evaluate the involvement of inflammasome activation in Cav-1-mediated protection from bleomycin-induced fibrosis, we measured expression of cleaved caspase-1 and IL-1β in mouse lungs 21 days after treatment with bleomycin, with or without Cav-1gene transfer. Increased caspase-1 activation and production of mature IL-1β were observed in bleomycin alone and GFP vector control groups (Fig. 5). Gene transfer of Cav-1 significantly reduced the expression of cleaved caspase-1 and IL-1β in response to bleomycin but had no effect in the absence of bleomycin. These results collectively indicate that gene transfer of Cav-1 downregulates activation of the inflammasome and downstream mediators induced by bleomycin.

### Inflammasome activation is involved in IPF patients with decreased Cav-1 expression

**Figure 6:**
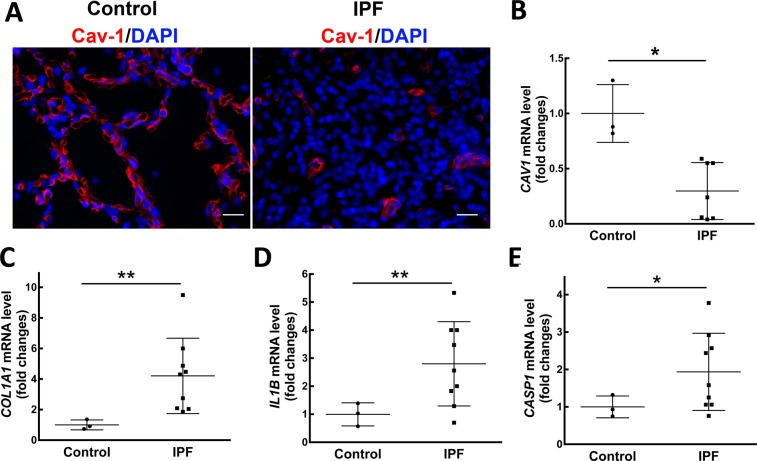
Inflammasome activation is involved in IPF patients with decreased Cav-1 expression. (**A**) Reduced Cav-1 expression was analyzed in lung sections by Immunofluorescence staining, compared to control (Cav-1, red; nucleus, blue). Representative photographs were taken at 400X magnification. Scale bar: 20 μm. mRNA levels of Cav-1 (**B**), collagen I (**C**), IL-1β (**D**) and caspase-1 (**E**) were detected by real-time PCR in lung tissue samples from IPF patients (n = 7–9) and control tissues (n = 3). Mean ± SD. Statistical analysis was analyzed by student *t* test. *P < 0.05 or **P < 0.01 compared to control.

Although the reduced expression of Cav-1 in patients with IPF that has been previously reported^26^, we determined whether inflammasome activation was altered in IPF patients with decreased Cav-1 expression. The reduction of Cav-1 protein expression was detected in lung sections of IPF patients by immunofluorescence staining and RT-PCR (Fig. 6A,B), consistent with previous studies^26^. We next analyzed collagen I expression by RT-PCR and observed a four-fold increase in IPF lung tissues compared with control tissues (Fig. 6B). To determine levels of inflammasome activation in the IPF lung, we examined mRNA levels of IL-1β and caspase-1 in IPF lung tissues by RT-PCR. A three-fold increase of IL-1β mRNA (Fig. 6C) and a two-fold increase of caspase-1 mRNA (Fig. 6D) were found in IPF lung tissues (n = 9), compared to control subjects (n = 3; p < 0.0087 or p < 0.0298, respectively).

### Overexpression of Cav-1 attenuates silica-induced inflammasome activation in primary epithelial type I cells

**Figure 7:**
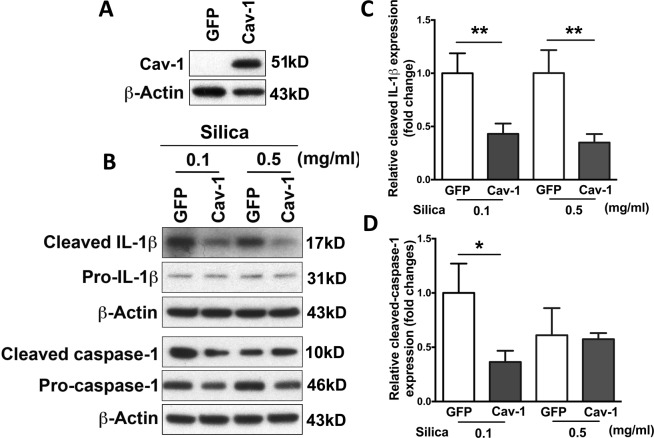
Overexpression of Cav-1 abolished silica-induced inflammasome activation in rat primary alveolar epithelial type I cells. Alveolar epithelial type II cells were isolated from naïve rat and transfected with Cav-1 or vector control. Four days later, primed for 5 hours with 1 mg/ml LPS, and then stimulated for 24 hours with silica. (**A**) Overexpression of Cav-1 was measured by western blot. Cropped blots were used in this figure. Original full-length blots are presented in Supplementary Figure S2A. (**B**) The presence of mature IL-1β and cleaved caspase-1 was analyzed in media supernatants, and pro-IL-1β and pro-caspase-1 were detected in cell extracts by western blot. Cropped blots were used in this figure. Original full-length blots are presented in Supplementary Figure S2B. The densitometry of western blot shows cleaved IL-1β (**C**) and cleaved caspase-1 (**D**). Statistical analysis was analyzed by student *t* test. *P < 0.05 compared to GFP control.

To study the relationship between Cav-1 gene transfer and inflammasome activation, we isolated primary epithelial type II cells from the lungs of naïve rats. It has been demonstrated previously that primary type II cells, cultured for several days, trans-differentiate to a type I cell phenotype^34–36^. Thus, cells were transfected with plasmids expressing GFP-tagged Cav-1 using electroporation after isolation and cultured on fibronectin-coated plates for 4 days followed by stimulation with lipopolysaccharide (LPS) and silica^37,38^. Pro-IL-1β is not constitutively expressed and requires induction in response to a stimulus, such as LPS. Silica is an activator of the inflammasome which is needed to cleave pro-caspase-1 to active caspase-1, which in turn mediates cleavage of pro-IL-1β to mature IL-1β. At first, successful transfection efficiency has been analyzed by western blot (Fig. 7A). As shown in Fig. 7B–D, treatment with crystal silica induced caspase-1 activation and released mature IL-1β in primary epithelial type I cells. However, overexpression of Cav-1 dramatically abolished the secretion of mature IL-1β as measured by immunoblot analysis of culture supernatants. Figure 7B also showed that the accumulation of the cleaved form of caspase-1 (p10) was attenuated in silica-stimulated cells after transfection with Cav-1, compared to GFP vector control. These data suggest that Cav-1 regulates inflammasome activation in lung epithelial cells.

## Discussion

Although the bleomycin mouse model has limitations, it is the most commonly used among current models for experimentally induced pulmonary fibrosis^39^. In the present study, we have demonstrated that electroporation-mediated gene transfer of Cav-1 to the lung protected from subsequent bleomycin-mediated fibrogenesis, attenuated recruitment of neutrophils and monocytes and/or macrophages, reduced activation of the inflammasome, and decreased deposition of collagen compared to controls. Overexpression of Cav-1 is capable of attenuating silica-induced activation of caspase-1 and maturation of pro-IL-1β in primary alveolar epithelial type I cells. We have also found that expression of IL-1β and caspase-1 was enhanced in IPF lung tissues compared with healthy controls, demonstrating that inflammasome activity plays a critical role in fibrogenesis and the pathogenesis of IPF. Taken together, our data indicate that electroporation-mediated gene transfer of Cav-1 protects from bleomycin-induced pulmonary fibrosis through downregulating inflammasome activity in the lung epithelium. These results help define the molecular mechanisms of IPF and develop a novel gene therapy approach for future clinical use.

IPF is a fatal disease with destruction of lung architecture and subsequent scar formation that ultimately leads to respiratory failure^1,2^. IPF results from repeated epithelial injury: immediately after injury, epithelial cells release inflammatory mediators that initiate the recruitment of inflammatory cells by increased permeability of the epithelial and endothelial barriers^1^. Neutrophils are quickly replaced by macrophages that produce various cytokines and chemokines. Activated alveolar epithelial cells induce fibroblast proliferation and differentiation into myofibroblasts. The latter further amplify the inflammatory responses that trigger myofibroblast transformation from epithelial-to-mesenchymal transition (EMT), bone marrow fibrocytes, and resident fibroblast proliferation. Finally, extracellular matrix (ECM) is secreted and apoptosis of epithelial cells is induced, causing IPF. Therefore, IPF is an epithelial-fibroblastic disease, in which unknown stimuli result in diffuse epithelial cell activation and aberrant epithelial cell repair^40^. Most studies for IPF have been focused on aberrant fibroblastic process, while it will need to have effective therapeutic strategies for IPF to target more than one of the pro-fibrotic pathways due to its complex pathogenesis^8^.

Recently, the FDA approved Ofev (nintedanib) and Esbriet (pirfenidone) to treat patients with IPF, each drug just slows, but does not reverse the progress of IPF^41,42^. Treatment effects of both Ofev and Esbriet were associated with a reduction of IL-1β levels in lung tissues from mouse models^43,44^. It indicates that inhibition of IL-1β might be a therapeutic target to dampen the progress of IPF. Although the innate immune system, such as alveolar macrophages and dendritic cells, are the primary source of the IL-1β and express high levels of inflammsome components, a growing body of evidence shows that lung epithelial cells are capable of activating NLRP3 and lead to IL-1β secretion in response to several stimuli. For example, mitochondrial ROS activates the NLRP3 inflammasome, leading to IL-1β secretion in bronchial epithelial cells^45^. Peeters and colleagues have also shown that the NLRP3 inflammasome exists and is functionally activated by crystalline silica in human lung epithelial cells^10,46^. Furthermore, alveolar epithelial cells cover more than 99% of the internal surface of the lung. They are the first cells to be exposed to a variety of danger signals and secrete pro-inflammatory cytokines including IL-1β in the pathogenesis of pulmonary fibrosis. Importantly, we also observed that mRNA levels of caspase-1 and IL-1β were significantly enhanced in lung tissues form IPF patients compared to controls^47^, which is consistent with previous studies in BAL of IPF^43,48^. This is data showing inflammasome activity is associated with the development of IPF, but it is hard to identify the cell types.

We observed that Cav-1 was highly expressed in alveolar epithelial type I cells in the normal lung, whereas expression of Cav-1 was greatly reduced in lungs from patients with IPF and bleomycin-induced fibrosis mouse model. The reduction of Cav-1 expression appeared in the areas with α-SMA positive staining. Therefore, treatments that increase expression of Cav-1 should be beneficial in pulmonary fibrosis. Wang *et al*. have reported that over-expression of Cav-1 by adenovirus can inhibit the pulmonary fibrotic response in a bleomycin-induced fibrosis mouse model through inhibition of TGF-β1-mediated production of collagen I and fibronectin by ERK and JNK pathway in fibroblasts^26^. Research from Tourkina’s group has demonstrated that systemic administration of the Cav-1 scaffolding domain peptide can block increased expression of collagen, tenascin-C and α-SMA by lung fibroblasts in a bleomycin-injured model. Its underlying mechanisms are associated with inhibition of MEK, ERK, JNK and Akt signaling in lung fibrosis^27^. Previous studies in fibrosis mouse models and in cultured alveolar epithelial cells have demonstrated that inflammasome activity and ultimately the secretion of active IL-1β stimulates the production of TGF-β to mediate the development of lung fibrosis^49,50^. Therefore, the inhibition of TGF-β production by reduced inflammasome activity might be involved in protective effect of Cav-1 gene transfer from bleomycin-induced fibrosis in this study.

Since Cav-1 is present in various cell types and plays a fundamental role in regulating multiple signaling pathways in the cells, the absence of Cav-1 leads to a significant increase of collagen expression and fibrosis, which is observed not only in the lung, but also in the other tissues including heart, vessel and skin^22,51^. Cav-1 conveys function in the development of fibrosis by regulating TGF-β1 signaling, tissue repair and cell proliferation, and inhibition of inflammation^51^. Currently, its therapeutic potential in fibroproliferative diseases is being considered. It is important to understand mechanisms by which Cav-1 is regulating the tissue reparative process for the understanding of fibrotic diseases and the development of their treatments. Given the majority of studies have demonstrated that Cav-1 has an anti-fibrotic effect in the lung^22–24,26,28^, a recent study has reported that Cav-1 deficient mice showed less fibrosis compared to wide type mice after bleomycin instillation^29^. However, research from the same group also found that Cav-1 deletion showed exacerbated cardiac fibrosis after cryoinjury, contrary to others findings^25,52^. Therefore, the role of Cav-1 in fibrotic disease and molecular mechanisms of IPF remain to be discovered.

Although using adenovirus and the Cav-1 scaffolding domain peptide are good approaches to determine the ability of Cav-1 to decrease fibrogenesis and pathogenesis of pulmonary fibrosis in an animal model, it would be beneficial to IPF patients that an approach can provide the ease and simplicity of administration, leading to safe, efficient and reproducible Cav-1 expression. Research from our laboratory has demonstrated that electroporation can be used to efficiently deliver DNA to the lung, resulting in high-level gene expression with no cardiac dysfunction and no damage, trauma, or inflammation in the lungs of mice^32,53,54^, rats^55^, and pigs^56^. Further, we have successfully used electroporation to deliver genes with high efficiency to 50 kg pigs, which are the size similar to humans, and have even used the technique to treat sepsis-induced acute lung injury^56^. Combined with the fact that there are currently 53 Phase I, Phase I/2, and Phase 2 clinical trials using electroporation for gene delivery to the skin and muscle, primarily to treat cancers and as vaccines^57^, our results here suggest that the combination of Cav-1 gene therapy and electroporation-mediated delivery could be clinically applicable to treat this otherwise refractive disease.

## Methods

### Human lung tissues

Healthy and fibrotic lung tissues were obtained from the NIH sponsored Lung Tissue Research Consortium (LTRC). Additional lung tissue was obtained from surgical lung biopsies performed at the University of Rochester using an Institutional Review Board approved protocol. Paraffin embedded human lung tissue sections were prepared as previously described after histopathological confirmation of the presence (UIP) or absence of pathology (healthy)^58^. Informed consent was obtained from all human subjects. All research was performed in accordance with relevant guidelines that had previously been reviewed and approved by an Institutional Review Board at the University of Rochester.

### Plasmids

The plasmid pUbC-GFP expresses GFP from the ubiquitin C (UbC) promoter (Addgene, Cambridge, MA). pCMV-Cav1 expresses a GFP- tagged mouse caveolae protein 1 (Cav1) from the CMV promoter (Origene, Rockville, MD), and pUbC-Cav1 expresses a GFP-tagged mouse Cav1 from the ubiquitin (UbC) promoter cloned from pUbC-GFP and pCMV-Cav1 plasmids. All plasmids were purified using Qiagen Giga-prep kits (Qiagen, Chatsworth, CA) and suspended in 10 mM Tris-HCl (pH 8.0), 1 mM ethylenediaminetetraacetic acid, and 140 mM NaCl.

### Animals

All animal usage was reviewed and approved by the University Committee on Animal Resources at University of Rochester. All experimental procedures were performed accordance with institutional guidelines for the care and use of laboratory animals in an American Association for the Accreditation of Laboratory Animal Care-approved facility. C57BL/6 J mice were purchased from Charles River Laboratories. Animals were fed with a normal rodent diet ad libitum.

### Silica preparation

Silica crystals (MIN-U-SIL-5) from US Silica (Dubberly, LA) were prepared as previously described^10,38^. Briefly, silica crystals were UV-irradiated overnight to inactivate possible contaminating endotoxin and silica suspensions were sonicated for 20 min and aspirated 6 times through a 26-gauge needle before they were added to cell culture. Silica crystals of 5 μm in length were used in all experiments.

### Cell culture, transfection and silica stimulation

Primary rat lung alveolar epithelial cells were isolated as previously described^36^. Cells in suspension were transfected by electroporation (280 V, 500 μF) after isolation and cultured in 6-well plates coated with 20 μg/ml fibronectin (Sigma-Aldrich, St. Louis, MO). All cells were cultured in Dulbecco’s modified Eagles medium (DMEM with high glucose, Cellgro, Manassas, VA) supplemented with 10% FBS, antibiotics and antimycotics (Gibco, Carlsbad, CA). Four days after transfection, cells were primed with 0.5 μg/ml of LPS (Sigma-Aldrich, St. Louis, MO) for 5 hours and then stimulated with 0.1 mg/ml or 0.5 mg/ml of silica for 24 hours.

### *In vivo* gene transfer and induction of pulmonary fibrosis

Male C57BL/6 mice (9–11 weeks) were anesthetized with isoflurane and 100 μg each of plasmids expressing Cav1 or GFP were delivered in 50 μl of 10 mM Tris-HCl (pH 8.0), 1 mM EDTA, and 140 mM NaCl, to mouse lungs by aspiration. Eight, 10 msec square wave pulses at a field strength of 200 V/cm were immediately applied using cutaneous electrophysiology electrodes (Medtronic, Redmond, WA) placed on the mouse chest with an ECM830 electroporator (BTX, Harvard Apparatus, Holliston, MA). All bleomycin-challenged mice received two units of bleomycin (Cayman Chemical Company, Ann Arbor, MI) per kg of body weight in 50 μl of phosphate-buffered saline (PBS) by aspiration, one day after gene transfer.

### Western blot analysis

Western blots were performed as previously described^59^. Briefly, lung tissues or cells were solubilized in lysis buffer containing protease inhibitor. Twenty μg of total protein was loaded on 12% SDS-PAGE, transferred to PVDF membrane, and probed with primary antibodies against Cav1 (Cell Signaling Technology, Danvers, MA), IL-1β (Cell Signaling Technology), caspase-1 (Santa Cruz Biotechnology, Dallas, TX) or β-actin (Sigma-Aldrich, St. Louis, MO). To detect inflammasome activation in cells, supernatants were collected and precipitated as described previously^60^. Supernatants were precipitated with 1 volume methanol, ¼ volume chloroform, and the precipitate was washed in 1 volume methanol and resuspended in 50 μl SDS loading buffer followed electrophoresis and transferring as above. Proteins were probed with primary antibodies against IL-1β and caspase-1. Data were analyzed using NIH Image J software.

### Histopathologic and immunhistochemical analysis

Lungs were perfused and inflated with 20 cc/kg aqueous buffered zinc formalin (Z-FIX; Anatech, Battle Creek, MI) immediately following euthanasia and used for paraffin-embedding. Sections (5 µm) were stained with hematoxylin and eosin and Masson’s trichrome, blinded, and reviewed for analysis of pathological changes in the lung according to our previous studies^59^. The severity of fibrosis was evaluated based on hematoxylin and eosin staining using the Ashcroft scale as previously described^61^. A fibrotic score (Ashcroft scale) was obtained as follows: the severity of the fibrotic changes in each lung section was given as the mean score from the observed microscopic fields. Each field was evaluated individually for fibrotic severity and allotted a score from 0 (normal) to 8 (total fibrosis). The fibrotic score for each field was averaged and presented as the average for each lung section.

### Bronchoalveolar lavage (BAL) analysis

BAL was performed as described previously^53^. Briefly, two separate 0.7 ml aliquots of sterile PBS were instilled into mouse lungs for lavage. The fluid was placed on ice for immediate processing and the total number of cells in the lavage was determined using a hemocytometer. Cells from the BAL were stained with Diff-Quik^TM^ (Siemens, Newark, DE) after cytospin.

### ELISA analysis

IL-1β levels in cells, mouse BAL, and lung homogenates were measured by ELISA according to the manufacturer’s instructions (Mouse DuoSet, R&D Systems, Minneapolis, MN).

### Quantitative RT-PCR

Total RNA was isolated from mouse or human lung tissue samples using Trizol reagent (Invitrogen, Carlsbad, CA). cDNA was prepared using Reverse Transcription System (Promega, Madison, WI) following the manufacturer’s instructions and quantitative RT-PCR was performed as previously described^59^. Real time PCR amplifications were performed using SYBR Green supermix (Bio-Rad). The relative quantities of mRNAs were obtained by the 2^-Δ^(^Δ^Ct) method and normalized with glyceraldehyde-3-phosphate dehydrogenase (GAPDH) housekeeping gene. The primer sequences are shown in Supplementary Table S1.

### Immunofluorescence staining

Immunofluorescence staining was performed as described previously^36^. Paraffin-embedded tissue sections (5 µm) were rehydrated and subjected to antigen retrieval in Tris-HCl buffer (100 mM, pH 9.5). Sections were immunostained overnight with FITC conjugated anti-GFP antibody (Abcam, Cambridge, MA), rabbit anti-Caveolin-1 antibody (Cell Signaling Technology, Danvers, MA), mouse anti-alpha smooth muscle actin antibody (DAKO, Carpinteria, CA), hamster anti-T1α antibody (Developmental Studies Hybridoma Bank at the University of Iowa, Iowa City, IA) as a marker for epithelial type I cells, and mouse anti-ABCA3 antibody (Seven Hills, Cincinnati, OH) as a marker for epithelial type II cells. The immune complexes were detected using Alexa Fluor conjugated secondary antibodies (Invitrogen, Grand Island, NY) before sections were counterstained with DAPI. Stained sections were visualized using a Leica DM RXA2 microscope (Leica, Wetzlar, Germany).

### Statistical analysis

Quantitative results are expressed as mean ± SEM for *in vivo* studies and mean ± SD for *in vitro* experiments. The data were evaluated statistically with one-way ANOVA or *t* test and *P*-values < 0.05 were considered statistically significant.

## Supplementary information

Caveolin-1 gene therapy inhibits inflammasome activation to protect from bleomycin-induced pulmonary fibrosis

## Data Availability

The data generated and analyzed during the present study are available from the corresponding author upon reasonable request.
