# Supplementary material for: Caveolin-1 gene therapy inhibits inflammasome activation to protect from bleomycin-induced pulmonary fibrosis
Source: Sci Rep. 2019 Dec 23;9:19643. doi: 10.1038/s41598-019-55819-y (PMC6928213; doi:10.1038/s41598-019-55819-y)
Supplement: Supplementary file 1 — Caveolin-1 gene therapy inhibits inflammasome activation to protect from bleomycin-induced pulmonary fibrosis [file 41598_2019_55819_MOESM1_ESM.pdf]

Caveolin-1 gene therapy inhibits inflammasome activation to protect from bleomycin-induced pulmonary fibrosis

Xin Lin[1], Michael Barravecchia[1], R. Matthew Kottmann[2], Patricia Sime[2] & David A Dean[1]*

[1]Department of Pediatrics, School of Medicine and Dentistry, University of Rochester, Rochester, New York 14642, USA. [2]Department of Medicine, School of Medicine and Dentistry, University of Rochester, Rochester, New York 14642, USA

*Correspondence and request for reprint should be addressed to D.D. (email: david_dean@urmc.rochester.edu). Department of Pediatrics, University of Rochester, 601 Elmwood Avenue BOX 850, Rochester, NY 14642, USA

Supplementary Table S1. Primers used for RT-PCR

Name	Sense	Antisense
Mouse Cav-1	5'-CATCTCTACACTGTTCCCATCC-3'	5'-TGTGCGCGTCATACACTT-3'
Mouse GAPDH	5'-TGTGATGGGTGTGAACCACGAGAA-3'	5'-GAGCCCTTCCACAATGCCAAAGTT-3'
Human Collagen I	5'- CCCTAACCAAGGATGCACTATG-3'	5'-CAGTTCTTGGCTGGGATGTT-3'
Human Caspase-1	5'- GGATAAGACCCGAGCTTTGATT-3'	5'-CTGCCAGGTAACTGTCTTCTTC-3'
Human IL-1β	5'-GGATAAGACCCGAGCTTTGATT-3'	5'-CTGCCAGGTAACTGTCTTCTTC-3'
Human Cav-1	5'-CTTCGCCATTCTCTCTTTCCT-3'	5'-GGTGTGGACGTAGATGGAATAG-3'
Human GAPDH	5'-GGTGTGAACCATGAGAAGTATGA-3'	5'-GAGTCCTTCCACGATACCAAAG -3'

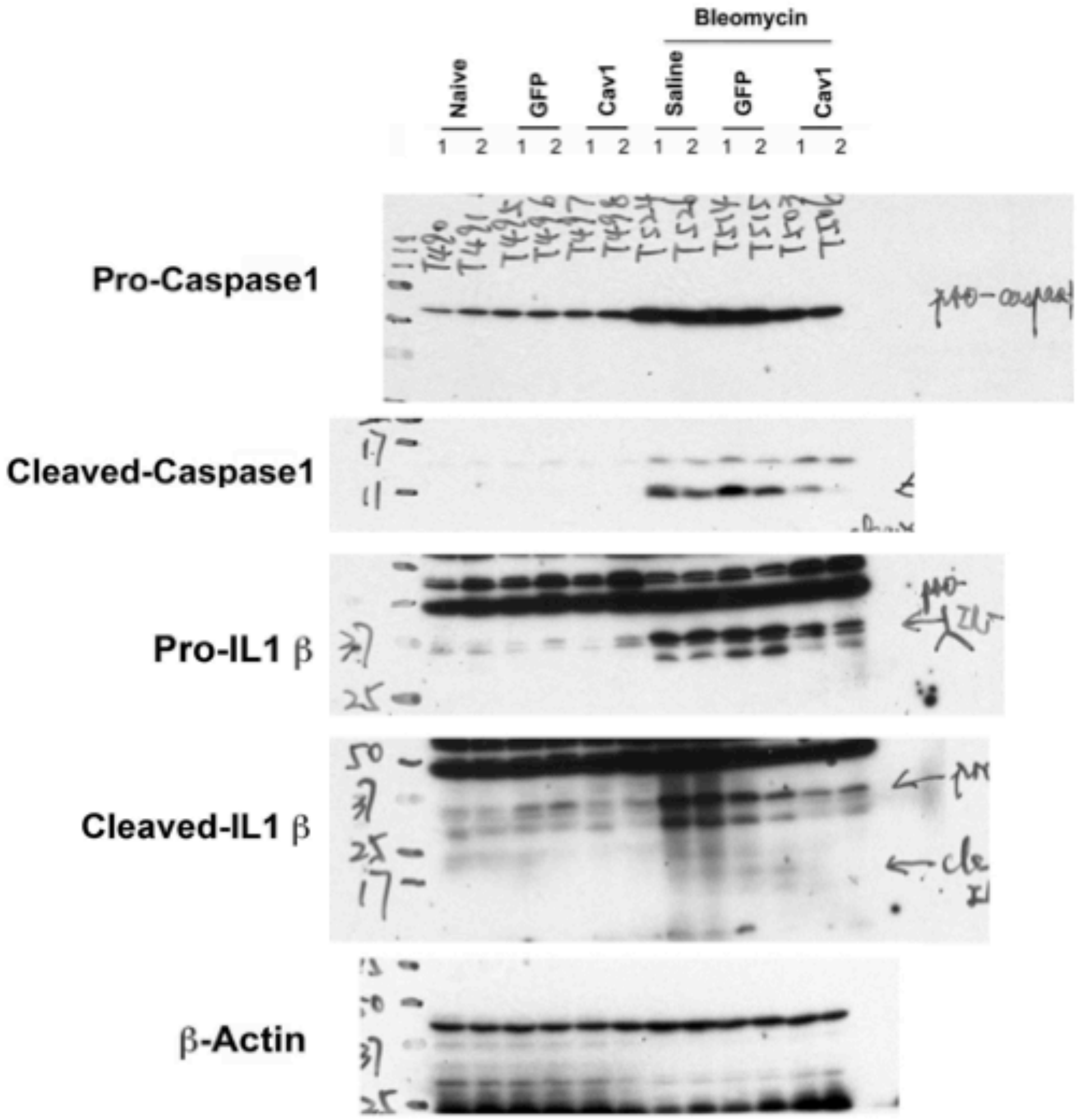

Supplementary Figure S1. Original full-length blots used in Figure 5 are shown.

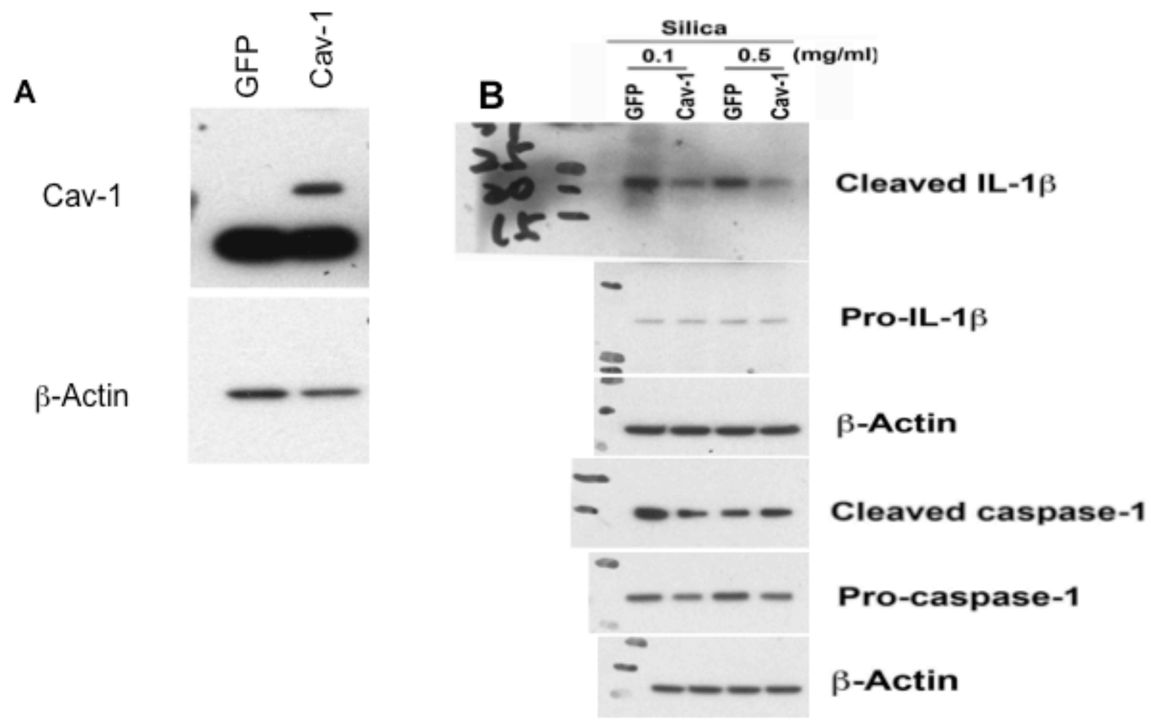

Supplementary Figure S2. Original full-length blots used in Figure 7 are shown.
